# School Teachers' Knowledge and Attitudes Related to Anaphylactic Reactions: A Cross-Sectional Study

**DOI:** 10.7759/cureus.72189

**Published:** 2024-10-23

**Authors:** Maan Jamjoom, Bsaim A Altirkistani, Rahaf A Hubayni, Jamil M Baljoon, Nooran S Felemban, Raghad A Aldahhas, Haitham M Alghamdi, Razan A Altirkistani

**Affiliations:** 1 College of Medicine, King Saud Bin Abdulaziz University for Health Sciences, Jeddah, SAU; 2 College of Medicine, King Abdullah International Medical Research Center, Jeddah, SAU; 3 Emergency Medicine Department, King Abdulaziz Medical City, Jeddah, SAU; 4 Pediatrics, King Abdullah Specialist Hospital, Jeddah, SAU; 5 Pediatric Emergency, King Abdulaziz Medical City, Jeddah, SAU; 6 Pediatrics, Al Aziziyah Children Hospital, Ministry of Health, Jeddah, SAU; 7 Nursing, King Abdullah Medical City, Makkah, SAU

**Keywords:** allergy, anaphylaxis, emergency, epinephrine, pediatric

## Abstract

Introduction: Anaphylaxis is a severe allergic reaction marked by a sudden onset of symptoms affecting multiple bodily systems, and if not addressed promptly, it can lead to fatal outcomes. The primary clinical manifestations often involve skin rash, respiratory distress, and cardiovascular symptoms. Identifying these signs early is crucial for timely intervention, including the administration of epinephrine, aiming to prevent loss of life. This study aims to assess the awareness of symptoms and management of anaphylactic reactions among teachers in Jeddah/Makkah, Saudi Arabia.

Methodology: This cross-sectional study included 742 teachers from both governmental and private schools in Jeddah/Makkah, Saudi Arabia. Only those schools who agreed and facilitated the process of distributing the questionnaire were included in which an electronic structured questionnaire was utilized and distributed among participants. Statistical analysis was done using JMP Pro 15 software (JMP Statistical Discovery LLC, Cary, NC).

Results: Most participants (n = 501, 67.5%) were female, and 374 (50.4%) were between 40 and 50 years old. Most participants believed that skin and eye manifestations were the most common clinical presentation. When inquired about anaphylaxis treatment, the majority of teachers (57%) opted for antihistamines as the preferred emergency treatment. Surprisingly, 407 of the 742 (54.9%) participants have never heard of epinephrine injections.

Conclusion: This study concluded that the knowledge of anaphylactic reactions among teachers in Saudi Arabia is limited. Therefore, educational campaigns and programs about anaphylaxis and the management of allergic reactions are recommended to elevate awareness within the teaching community, contributing to a more informed and prepared educational environment.

## Introduction

Anaphylaxis is an acute and severe allergic reaction that rapidly progresses to a life-threatening condition [1]. It can be fatal if left untreated as it results in cardio-respiratory collapse. Anaphylaxis is characterized by a multisystem acute onset of symptoms. The clinical symptoms mainly depend on the mode of allergen exposure. Generally, anaphylaxis can present with pruritus, urticarial, and respiratory manifestations [2]. The most frequent symptoms are skin rash, followed by the involvement of the respiratory and cardiovascular systems [3]. However, anaphylaxis can develop in the absence of skin changes. Moreover, respiratory and cardiovascular signs/symptoms are the most life-threatening features that might lead to death [4].

The lifetime prevalence of anaphylaxis has been estimated between 1% and 3%, though it is becoming more prevalent [2]. In the United States of America, anaphylaxis happens in 30 of 100,000 individuals per year, and the reported death rate was 1%-2% [5]. Anaphylaxis may occur at any age [2]. There is an increase in the number of cases of anaphylaxis that require visiting pediatric emergency departments [6]. One in 10,000 children has an anaphylactic event per year. Approximately 82% of these episodes occur in school-age children [7]. Most anaphylaxis attacks in the pediatric age group occur in the presence of family members or at school [6]. For instance, a study showed that 20%-25% of children experience their first anaphylactic reaction in school [8]. Anaphylaxis in school-age children is mainly caused by specific types of foods, such as peanuts and cow’s milk, in addition to insect bites, medications, latex rubber, and exercise [9]. As mentioned before, it is estimated that 20.0% of anaphylactic reactions in children occurred while they were in school. A total of 30.0% of these affected children had an allergic reaction for the first time or had a known allergy, but the school staff were unaware [10]. To avoid consequences, all schools must be prepared and trained to manage unexpected anaphylactic events.

The first-line treatment of anaphylaxis is the immediate administration of epinephrine. A case series study done in the United States reported that nine out of 32 fatalities that occurred in school were associated with delays in administering epinephrine [11]. In schools, they may use an epinephrine auto-injector as it is easier for non-medical personnel [12]. Numerous studies have reported that school personnel globally are primarily unprepared and unaware of anaphylactic events happening in schools. Locally, in Saudi Arabia, one study was conducted in the Qassim region to assess the awareness of anaphylaxis among school teachers. Consistent with international studies, the results showed poor awareness and knowledge regarding anaphylaxis. This study aims to assess the awareness of symptoms and management of anaphylactic reactions among teachers in Jeddah/Makkah, Saudi Arabia [12].

## Materials and methods

Study design, settings, and IRB details

This research is a cross-sectional study conducted in schools in Jeddah/Makkah, Saudi Arabia. All procedures performed in this study involving human participants were in accordance with the ethical standards of the institutional and/or national research committee and the 1964 Helsinki Declaration and its later amendments or comparable ethical standards. The study was approved by the IRB Committee at King Abdullah International Medical Research Center (KAIMRC).

Study population and inclusion and exclusion criteria

Only those governmental and private schools that agreed and facilitated the process of distributing the questionnaire were included and considered in this research. While school students or teachers in their initial training phase were excluded.

Questionnaire tool, source, development, and validation

The appropriateness and fitness of the questionnaire were ensured by structuring the elements of the questionnaire thoroughly by the research team. Content validity and a pilot study were applied to determine the clarity of the questionnaire and the relevance of the questions in relation to the research objective. Appropriate adjustments were performed as necessary.

Data collection and tool administration technique

This study recruited data collectors to distribute the electronic questionnaire to the targeted population by visiting in person the schools that provided their facilitation of the process. A non-probability convenience technique was applied in this study. Data collection took place from November 2023 to February 2024.

Outcome variable and data

The first part of the questionnaire was the informed consent to ensure the participants’ acceptance to participate in the research and clarify that participating in this research is optional and that withdrawal from the research is permitted in any phase. No personal information was needed, and their participation was anonymous. First, demographical information, such as age, gender, and education, was asked and collected. Next, this study involved questions about knowledge of anaphylaxis to achieve the aim of the study, such as the most common age of anaphylaxis and symptoms of anaphylaxis shock. Subsequently, the teachers’ attitude regarding anaphylaxis was assessed by asking them questions related to actions they would perform if they encountered an anaphylactic reaction in school. Those teachers who reported that they dealt with an anaphylactic incident were asked about actions made, the resulting outcome of the incident, and whether there was supervision or not.

Statistical analysis

Statistical analysis was done using JMP Pro 17.1 software (JMP Statistical Discovery LLC, Cary, NC). We used a chi-square test to measure our variables. To find associations between variables, Pearson’s test and likelihood ratio were used, and a p-value of less than or equal to 0.05 was considered significant.

## Results

Demographic characteristics of the participants

A total of 742 school teachers agreed to participate in this study to evaluate their knowledge and attitudes related to anaphylactic reactions. Of the participants, 501 (67.5%) were females, while the remaining 241 (32.5%) were males. The single highest age demographic was teachers between the ages of 40 and 50 years, which constituted 374 of the 742 participants (50.4%). On the other hand, the least age demographic in this study were teachers younger than 23 years old, with 12 out of 742 (1.6%). Of the 742 teachers who agreed to participate in this study, 688 (92.7%) were Saudi nationals, and 586 (79%) were married. Regarding the level of education of the 742 participants, 630 (84.9%) had a university degree, 58 (7.8%) had a diploma degree, and the remaining 54 (7.3%) participants had a master’s degree. The three most common specialties of the 742 teachers were the following: science with a number of 153 (20.6%) participants; Islamic studies with 108 (14.6%) participants; and Arabic language with 95 (12.8%) participants, as depicted in Table 1.

**Table 1:** The demographic characteristics of the study participants.

Parameter	Value
Gender (%)	(n = 742)
Male	241 (32.5%)
Female	501 (67.5%)
Age (%)	
Less than 23 years old	12 (1.6%)
23-29 years old	66 (8.9%)
30-39 years old	170 (22.9%)
40-50 years old	374 (50.4%)
More than 50 years old	120 (16.2%)
Marital status (%)	
Married	584 (79%)
Single	96 (12.9%)
Divorced	46 (6.2%)
Widow	14 (1.9%)
Nationality (%)	
Saudi	688 (92.7%)
Non-Saudi	64 (7.3%)
Family income per month (%)	
Less than 4000 Saudi Riyals	50 (6.7%)
4000-10000 Saudi Riyals	208 (28%)
11000-20000 Saudi Riyals	398 (53.6%)
More than 20000 Saudi Riyals	86 (11.6%)
Level of education (%)	
Diploma	58 (7.8%)
University	630 (84.9%)
Master’s degree	54 (7.3%)
Specialty (%)	
Science	153 (20.6%)
Islamic studies	108 (14.6%)
Arabic language	95 (12.8%)
English language	72 (9.7%)
Social studies	67 (9%)
Math	65 (8.8%)
Years in service (%)	
Less than 5 years	90 (12.1%)
5-10 years	147 (19.8%)
11-15 years	162 (21.8%)
More than 15 years	343 (46.2%)

Knowledge, attitude, and experience of the participants with anaphylactic reactions

**Figure 1:**
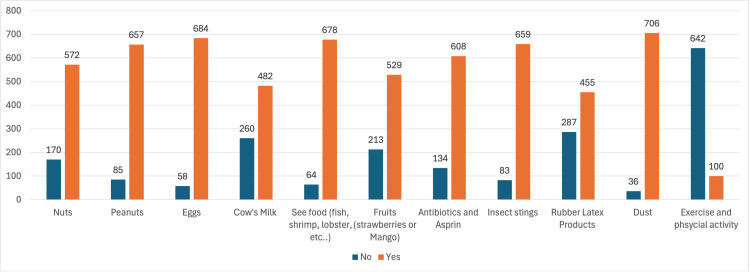
Factors that can trigger an anaphylactic reaction.

Among the 742 teachers surveyed for this study, 250 (33.7%) had never heard about anaphylaxis before. When the teachers were asked whether their school has first-aid medications for anaphylaxis, 245 (24.9%) answered no, 185 (24.9%) answered yes, while the remaining 312 (42.1%) participants did not know the answer. Furthermore, the most common “reason” that participants believe that it cannot cause anaphylaxis was exercise and physical activity since 642 of 742 (86.5%) assumed that it cannot cause anaphylaxis. The remaining answers were the following in descending order: 287 (38.7%) assumed that rubber latex products cannot cause anaphylaxis; 260 (35%) believed that cow’s milk does not cause anaphylaxis; 213 (28.7%) thought that fruits such as apples and mangos cannot cause anaphylaxis; 170 (22.9%) believed that nuts cannot cause anaphylaxis; 134 (18.1%) thought that antibiotics and aspirin cannot cause anaphylaxis; 85 (11.5%) thought that peanuts could not cause anaphylaxis; 83 (11.2%) believed that insect stings could not cause anaphylaxis; 64 (8.6%) believed that sea foods such as fish, shrimp, lobster, and shellfish could not cause anaphylaxis; 58 (7.8%) believed that chicken eggs could not cause anaphylaxis; while most participants believed that dust and pollens are a most likely product to cause anaphylaxis since 706 of 742 (95.2%) agreed that it could cause anaphylaxis, and only 36 (4.9%) assumed that it cannot cause anaphylaxis, as shown in Figure 1.

**Figure 2:**
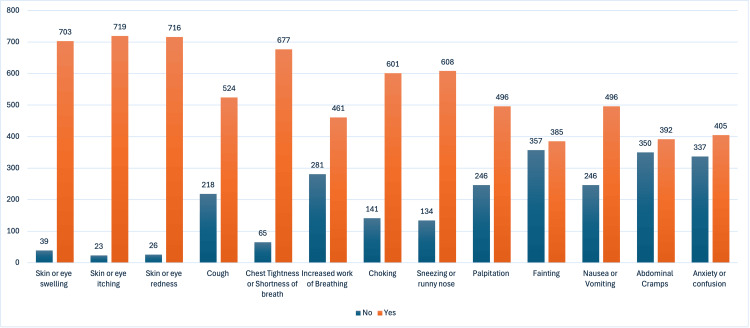
The teachers presume that the subsequent signs and symptoms are indicative of anaphylaxis.

In regard to signs/symptoms associated with anaphylaxis, participants believed that skin and/or eye itchiness and skin and/or eye redness were the two most common anaphylactic signs, with numbers of 719 (96.9%) and 716 (96.5%), respectively. The remaining answers are illustrated in Figure 2.

Anaphylaxis management and preparedness: treatment and protocols

Regarding information about the treatment of anaphylaxis, 349 of the 742 (47%) participants believe that the disappearance of symptoms after anaphylaxis is a reassuring sign that indicates the good health status of the student. Moreover, when the teachers were asked about the treatment of anaphylaxis, most participants chose antihistamines as the emergency treatment for anaphylaxis with a number of 432 out of 742 (57%). Other medications selected for treating anaphylaxis by the teachers were epinephrine, painkillers, and antiemetics, with a total number of 219 (29.5%), 90 (12.1%), and 10 (1.4%), respectively. Consequently, 407 of the 742 (54.9%) participants have never heard of epinephrine injections, and 121 (16.3%) of the teachers know how to use epinephrine injections. When teachers were asked about the route of administration for epinephrine in anaphylaxis, most participants answered “Subcutaneous” or “Intravenous,” with a number of 362 (48.8%) and 344 (46.4%), respectively. Other routes the participants selected were the oral route and inhalation, with a total number of 23 (3.1%) and 13 (1.8%), respectively. When participants were asked to choose the first action done in anaphylaxis, the most common action chosen was to “call 911 emergency service,” with a number of 236 of 742 (31.8%) participants. The other two common answers chosen by the participants were “Remove inciting allergen” and “Take him/her to hospital,” with similar numbers of 175 (23.6%) and 170 (22.9%), respectively. Among the 742 participants, 274 (36.9%) have attended first aid courses. Consequently, 451 of the 742 (60.8%) teachers believe that anaphylaxis reactions in children can put children’s health in danger. Also, only 109 (14.7%) and 20 (2.7%) participants rated their confidence in managing an anaphylaxis reaction as confident and very confident, respectively.

When asked about the age group that was most susceptible to anaphylaxis reaction, the most common answer was “I don’t know” since it constituted 242 (32.6%) of the participants. The second most common answer was three- to five-year-old children, with a number of 154 (20.8%). For the chosen reasons behind the lack of awareness about anaphylaxis reaction in the community, the answers were the following: 283 (38.1%) chose that the reason was a loss of interest of the community to learn first aid; 255 (34.4%) mentioned that due to lack of information from doctors and health campaigns; and the remaining 204 (27.5%) participants say that the cause is first aid is not mandatory in schools, as depicted in Table 2.

**Table 2:** Anaphylaxis management and preparedness: treatment and protocols.

Parameter	Value (n = 742)
Disappearance of symptoms indicates what after the incidence? (%)	
Reassuring, reflecting good health status	349 (47%)
Serious signs require immediate intervention	108 (14.6%)
Disappearance of symptoms does not reflect anything	40 (5.4%)
I do not know the answer	245 (33%)
Which of the following medications is used for treating anaphylaxis in emergency settings? (%)	
Antihistamines	423 (57%)
Epinephrine	219 (29.5%)
Painkillers	90 (12.1%)
Antiemetics	10 (1.4%)
Have you heard of epinephrine pen injection? (%)	
Yes	121 (16.3%)
No	621 (83.7%)
How should epinephrine be administered? (%)	
Subcutaneous	362 (48.8%)
Intravenous	344 (46.4%)
Orally	23 (3.1%)
Inhalation	13 (1.8%)
Which of the following should be the first action taken in anaphylactic cases? (%)	
Call 911 Emergency Services	236 (31.8%)
Removing inciting allergen	175 (23.6%)
Take the student to the hospital	170 (22.9%)
Call the student’s family	114 (15.4%)
Give the student an epinephrine pen injection	47 (6.3%)
Have you attended first aid courses? (%)	
Yes	274 (36.9%)
No	468 (63.1%)
How would you rate your level of confidence if you saw a student experiencing anaphylaxis? (%)	
Very confident	20 (2.7%)
Confident	109 (14.7%)
Neutral	261 (35.2%)
Unconfident	200 (27%)
Very unconfident	152 (20.5%)
How dangerous can anaphylaxis be in children? (%)	
It puts the child’s health in danger	451 (60.8%)
Mildly dangerous	123 (16.6%)
Does not cause any danger	25 (3.4%)
I do not know	143 (19.3%)
At which age is anaphylaxis reaction commonly occurring? (%)	
Less than 1 year old	107 (14.4%)
1-2 years old	136 (18.3%)
3-5 years old	154 (20.8%)
6-8 years old	63 (8.5%)
More than or equal to 9 years old	40 (5.4%)
I do not know	242 (32.6%)
In your opinion, why there is a lack of awareness about anaphylactic reactions in the community? (%)	
Loss of interest of the community to learn about first aid	283 (38.1%)
Lack of information from doctors and health campaigns	255 (34.4%)
First aid is not mandatory in school	204 (27.5%)

The preferred sources for teachers to acquire medical knowledge based on their preferences

**Figure 3:**
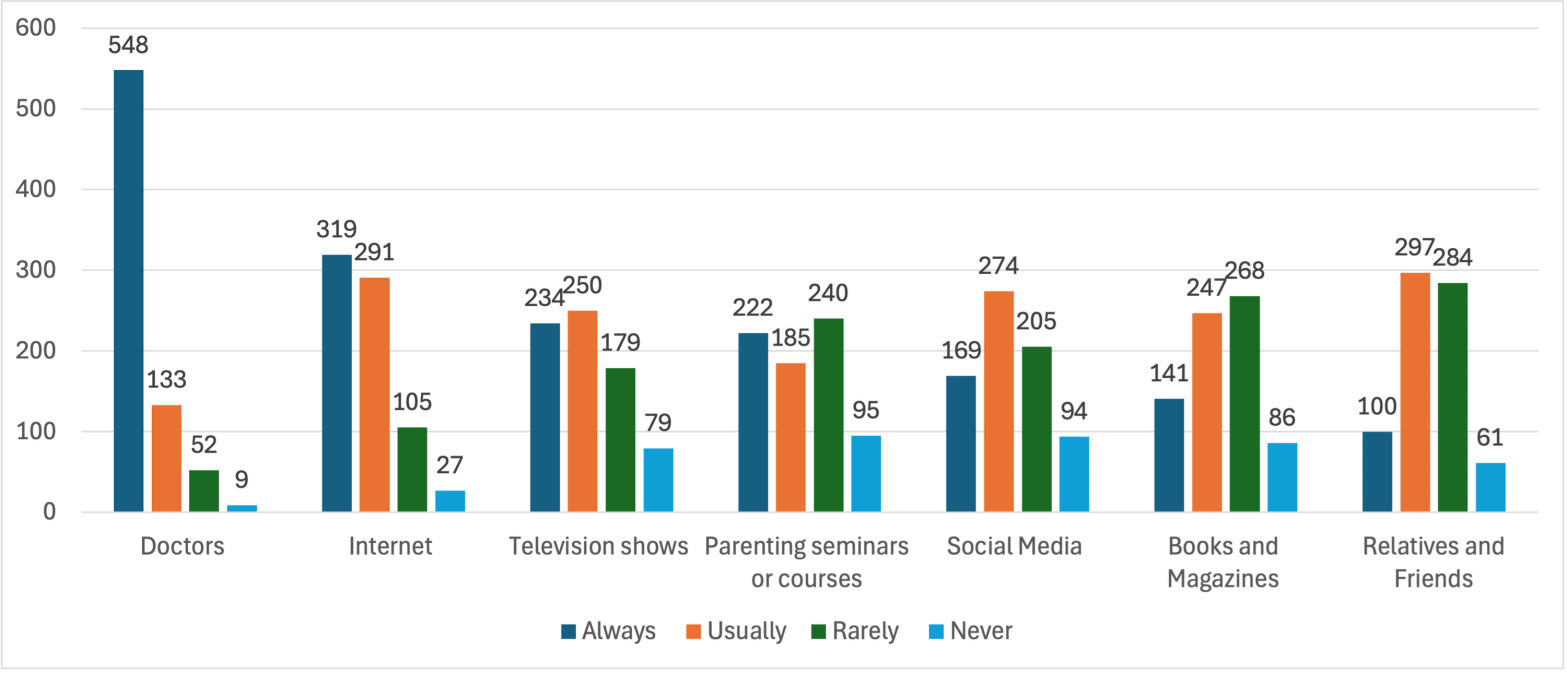
The preferred sources for teachers to acquire medical knowledge based on their preferences.

Most participants (548, 73.9%) reported that they always depend on a doctor as a medical source. On the other hand, 297 (40%) said they can usually depend on relatives and friends to get medical knowledge. Moreover, the most common answer for getting medical knowledge from “Books and Magazines” was “rarely,” with a number of 268 (36.1%). Likewise, 240 (32.4%) of the participants mentioned that they rarely get medical information from “Parenting seminars/courses.” In addition, 319 (43%) of the teachers who participated in this study said that they always depend on internet websites to get medical knowledge, and 274 (36.9%) mentioned that they get medical information from social media. Interestingly, 250 (33.7%) and 234 (31.5%) of the participants mentioned that they “usually” and “always” get medical knowledge from television shows, respectively, as illustrated in Figure 3.

Witnessing an anaphylactic reaction among school students in the workplace

Among the 742 teachers who answered this survey, 172 (23.2%) have witnessed an anaphylactic reaction in the workplace, 92 (53.5%) of which were female students. Among the witnessed anaphylactic reactions, 69 (40.1%) were not under surveillance when the anaphylactic reaction happened. Moreover, 89 (51.7%) were managed in school, while 63 (36.6%) were treated in the emergency room. Of the 172 cases, only six (3.5%) resulted in serious complications, as depicted in Table 3.

**Table 3:** Witnessing an anaphylactic reaction among school students in the workplace.

Parameter	Value (n = 172)	
Gender of the student who had anaphylactic shock (%)		
Male		80 (46.5%)
Female		92 (53.5%)
Was the student under surveillance while the anaphylaxis happened? (%)		
Yes		103 (59.9%)
No		69 (40.1%)
How was the incident dealt with? (%)		
The condition was managed in school		89 (51.7%)
The condition was managed and treated in the emergency room		63 (36.6%)
No action was taken		11 (6.4%)
Required admission to a hospital		9 (5.2%)
What was the outcome of the incident? (%)		
Recovery		97 (56.4%)
Required management by a doctor		69 (40.1%)
Serious complications		6 (3.5%)

A comparison between the level of education and knowledge about anaphylaxis

There is a significant difference between the level of education and knowing whether insect stings cause anaphylaxis. Among the 630 teachers who have a university degree, 568 (90.2%) believe that insect stings can cause anaphylaxis. However, only 46 of 54 (85.2%) of the teachers with a master's degree and 45 of 58 (77.6%) teachers with a diploma believe that insect stings can cause anaphylaxis. There was no significant difference between the level of education and the knowledge of the other 10 possible triggers for anaphylaxis. There was a significant difference between the level of education and whether "anxiety and confusion" are signs of anaphylaxis. Of the 54 teachers who graduated with a master's degree, 32 (59.3%) believe that "anxiety and confusion" can be a sign of anaphylaxis. In contrast, only 351 of 630 (55.7%) of university graduates believe that "anxiety and confusion" is a sign of anaphylaxis. Furthermore, 22 of 58 (37.9%) diploma graduates believe that "anxiety and confusion" is a sign of anaphylaxis. There was no significant difference when the level of education was compared to the other aforementioned symptoms.

**Figure 4:**
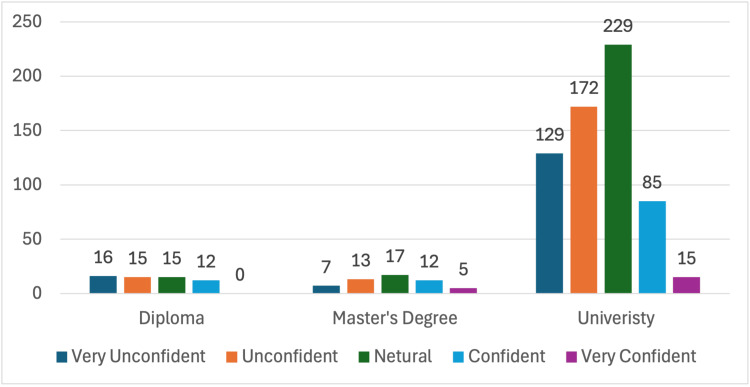
The level of confidence for dealing with an anaphylaxis case based on the level of education.

There is a significant difference between the level of confidence in dealing with a case of anaphylaxis and the level of education. Among the 54 teachers who have a master's degree, five (9.3%) and 12 (22.2%) believe that they are "Very confident" and "Confident" when dealing with an anaphylaxis case, respectively. Similarly, 15 out of 630 (2.4%) and 85 out of 630 (13.5%) of university graduate teachers believe that they are "Very confident" and "Confident" in dealing with an anaphylaxis case, respectively. On the other hand, 12 of 58 (20.7%) diploma graduates believe that they are "Confident" when dealing with anaphylaxis, whereas no teacher (0%) believes that they are "Very confident" in this matter, as illustrated in Figure 4.

Regarding the source of medical information, there was a significant difference between the teachers' education level and doctors being the source of medical information. Of the 54 teachers who had a master's degree, 32 (59.3%) "always" used doctors to acquire medical knowledge, whereas 15 (27.8%) "usually" used doctors to acquire medical information. For teachers who graduated from universities, 464 (73.7%) and 113 (17.9%) "always" and "usually" use doctors to get medical information, respectively. However, of the 58 teachers who have a diploma, 52 (89.7%) "always" use doctors to get medical knowledge. There was no significant difference when the level of education was compared to the other sources for medical knowledge, including relatives and friends, books and magazines, internet websites, social media, television programs, and parenting seminars/courses.

## Discussion

This survey provides valuable insights into teachers' knowledge, attitudes, and experiences regarding anaphylactic reactions among students. This study's findings suggest a mixed level of awareness and preparedness among the surveyed teachers. After reviewing local data, a cross-sectional study conducted in Al-Qassim in 2019 reported that teachers needed a better knowledge of anaphylaxis. Similarly, a systematic review that included 12 studies conducted in countries like Canada, the United States of America (USA), Australia, and Europe also reported poor knowledge of anaphylaxis and management. When assessing the demographics of the teachers who answered our survey, most participants were females (67.5%). Half of the teachers were between 40 and 50 years old, and most had university degrees. However, a study by Alsuhaibani et al. reported that 47.1% of participants were females, and 77.7% had completed their college studies. Also, they stated that women with bachelor's degrees have higher mean knowledge and attitude scores. Firstly, in the current study, the survey showed that a majority of teachers (57.7%) reported that at least one student in their school had a type of allergy. On the other hand, it is concerning that a significant proportion (33.7%) have never heard about anaphylaxis before. While in Al-Qassim, only a quarter of the teachers were aware of students with anaphylaxis. Internationally, the percentage of awareness of students with allergic reactions ranged from 3.5% in Spain to 52% in Turkey. This lack of awareness among teachers can be a critical issue, as prompt recognition of the anaphylaxis symptoms and appropriate immediate response are essential in managing anaphylactic reactions in schools where most schools do not have medical school clinics to provide prompt actions [12].

Understanding the symptoms and causes of anaphylaxis is crucial to effectively prevent adverse events of allergic reactions, especially in an educational setting. The survey also assessed the teachers' knowledge of potential triggers for anaphylaxis. The majority of teachers (95%.2) were aware that dust and pollen can cause anaphylaxis. However, a significant number of participants had misconceptions about other triggers such as rubber latex products, cow's milk, fruits, nuts, insect stings, antibiotics, and other substances that could potentially cause anaphylactic reactions. Moreover, the findings related to recognizing anaphylactic signs and symptoms are encouraging and concerning. Many teachers correctly identified common symptoms such as skin and/or eye itchiness, redness, swelling, and shortness of breath. On the other side, there were notable gaps in knowledge, with a considerable number of participants associating anaphylaxis with symptoms like sneezing/runny nose, cough, palpitations, fainting, nausea and/or vomiting, and abdominal cramps, which are not typical presentations of anaphylactic reactions. Likewise, Alsuhaibani et al. reported that the majority of teachers believed that the most common symptoms of anaphylaxis are shortness of breath, itching, and skin rash. Also, the most common causes were insect bites, pollens, and drugs [12]. One published paper in a tertiary center in Saudi Arabia assessed the prevalence of triggers and clinical severity associated with anaphylaxis in 2018. It stated that the most common symptoms were urticaria, angioedema, and shortness of breath, while the most common triggers of anaphylaxis were food, insect bites, drugs, and environmental causes [13].

This study shows that nearly half of the participants mistakenly believe that the disappearance of symptoms following anaphylaxis indicates good health status. This misconception could lead to delayed or inadequate treatment, as it overlooks the possibility of delayed or recurrent symptoms and the need for continued medical monitoring. Additionally, the majority of teachers identified antihistamines as the primary emergency treatment for anaphylaxis. While antihistamines can help with mild allergic reactions, they are not effective in addressing the life-threatening symptoms of anaphylaxis and are not recommended for immediate management. Using antihistamines before administering adrenaline can potentially delay the crucial first-line treatment. Adrenaline is prioritized for its rapid onset of action and more suitable pharmacological response [14,15]. The insufficient awareness of epinephrine's role as the primary treatment emphasizes the critical necessity for educational campaigns targeting educators. The research findings indicate deficiencies in participants' understanding and preparedness concerning anaphylaxis management. To illustrate, a considerable portion of respondents (54.9%) have never heard about epinephrine injections, the primary treatment for anaphylaxis, while only a minority (16.3%) know how to administer them. Additionally, this study shows that most participants did not select the appropriate administration route, i.e., intramuscular injection. A similar finding was demonstrated in a study conducted in the Al-Qassim region in Saudi Arabia, reporting that only 6% of subjects selected intramuscular epinephrine injections [12]. The relatively low participation in first aid courses further emphasizes the ongoing need for education and training in anaphylaxis management. Providing educational programs, as demonstrated by the minority who have attended first aid courses, can significantly enhance the teacher's knowledge and practice toward anaphylaxis [16]. Lastly, the acknowledgment among teachers of the severity of anaphylaxis, as indicated by the high percentage (60.8%) who believe it is endangering children's health, contrasts with their low confidence levels in managing anaphylaxis reactions. This highlights the discrepancy between their awareness and practical skills and emphasizes the importance of bridging this gap through comprehensive education and training.

The survey results reveal varying levels of awareness among teachers regarding the age group susceptible to anaphylaxis reactions. A concerning proportion (32.6%) answered "I do not know," and only (20.8%) answered from three to five years. The sources of medical knowledge range from traditional avenues like doctors to more contemporary platforms like the internet and social media, which is consistent with Alsuhaibani et al.'s findings [12]. Targeted interventions can enhance teachers' awareness and knowledge, fostering a more informed and proactive response to anaphylactic reactions within educational settings and the broader community.

In our study, we found that 23.2% of teachers had witnessed anaphylactic reactions among students in their schools. Research conducted in Saudi Arabia showed an almost similar percentage, with 28.6% of respondents having observed anaphylaxis [12]. This highlights the significance of addressing this condition within schools. However, in Spain, only 3.5% of teachers had encountered anaphylactic reactions among their students, suggesting some potential variations in prevalence rates among different regions [17,18]. Moreover, it is alarming that 40.1% of anaphylactic events happened without student supervision. This supports the need for improved surveillance procedures and highlights how crucial it is to maintain continuous vigilance on students who are at risk of anaphylaxis.

Additionally, the fact that half of anaphylactic cases (51.7%) are handled in schools raises questions about the effectiveness of in-school emergency response protocols. This highlights the necessity for schools to have trained staff, awareness of emergency medications like epinephrine, and work effectively with emergency services to achieve optimal outcomes for students who experience anaphylaxis when there is no doctor around in school. The majority of witnessed anaphylactic reactions in this study did not result in serious complications. This may suggest that prompt detection and intervention - whether in school or emergency services - were successful in preventing negative outcomes. However, it is often recommended to seek additional medical care and follow-up to monitor for any adverse events.

The findings highlight a significant association between teachers' educational background and their comprehension and confidence in managing anaphylaxis. While the majority of teachers at all educational levels acknowledge insect stings as potential triggers for anaphylaxis, there is a slight variance in awareness among different educational groups. To illustrate, among participants with university degrees, a large majority (90.2%) believe insect stings can cause anaphylaxis. However, among those with master's degrees, a slightly smaller percentage (85.2%) hold this recognition, and among those with diplomas, an even lower percentage (77.6%) share the same belief. Additionally, there is a clear correlation between the level of education and the recognition of some symptoms of anaphylaxis, particularly "anxiety and confusion." The results reveal that a higher proportion of teachers with master's degrees (59.3%) recognize "anxiety and confusion" as signs of anaphylaxis compared to university graduates (55.7%) and diploma graduates (37.9%). This suggests that higher educational levels may contribute to a better understanding of specific symptoms associated with anaphylaxis.

Furthermore, the data show that among individuals with master's degrees, a lower proportion feel "very confident" (9.3%) or "confident" (22.2%) in handling anaphylaxis cases compared to university graduates (2.4% and 13.5%, respectively) and diploma graduates (0% and 20.7%, respectively). This suggests that possessing higher levels of formal education does not necessarily lead to increased confidence levels in managing anaphylaxis conditions. Moreover, the results regarding teachers' sources of medical information underscore a notable correlation between their educational levels and their reliance on doctors for obtaining medical knowledge. Specifically, the data reveal that a more significant proportion of teachers with lower levels of formal education, such as diploma holders, consistently seek medical information from doctors compared to those with higher levels of education. For example, among teachers holding master's degrees, 59.3% consistently rely on doctors for medical knowledge, whereas this percentage rises to 73.7% among university graduates and further increases to 89.7% among diploma holders. This indicates that educators with lower levels of formal education exhibit a strong dependence on medical professionals as their primary source of medical information.

Limitations and recommendations

There are some limitations in this study. Specifically, it was conducted in Jeddah/Makkah only and many schools did not want to participate and involve their teachers in filling out the survey, which may affect the generalizability of the findings. The convenience sampling method used in our study has the potential for sampling bias. Thus, it is recommended to include other regions in Saudi Arabia to obtain more reliable outcomes. Consequently, further research is needed to understand the results of this study.

## Conclusions

This study concluded that the knowledge of anaphylactic reactions among teachers in some of Jeddah/Makkah schools is limited. Therefore, educational campaigns and programs about anaphylaxis and the management of allergic reactions are recommended to enhance awareness among teachers. In addition, health policies regarding anaphylaxis can be implemented in schools, such as providing schools with epinephrine injections, labeling children who have allergies, and training teachers to prevent serious complications.
